# 588. Ceftolozane/Tazobactam Susceptibility of Carbapenem-Resistant *Pseudomonas aeruginosa* and Carbapenem-Resistant *Enterobacterales* Isolates in a Tertiary Hospital in Korea

**DOI:** 10.1093/ofid/ofad500.657

**Published:** 2023-11-27

**Authors:** Kye-Hyung Kim, Seulgi Moon, Mee Kyung Ko, Jongyoun Yi

**Affiliations:** Pusan National University School of Medicine, Pusan, Pusan-jikhalsi, Republic of Korea; Department of Laboratory Medicine, Pusan National University School of Medicine, Busan, Pusan-jikhalsi, Republic of Korea; Pusan National University Hospital Biomedical Research Institute, Busan, Pusan-jikhalsi, Republic of Korea; Department of Laboratory Medicine, Pusan National University School of Medicine, Busan, Pusan-jikhalsi, Republic of Korea

## Abstract

**Background:**

Carbapenem-resistant *Enterobacterales* (CRE) and carbapenem-resistant *Pseudomonas aeruginosa* (CRPA) are significant nosocomial pathogens that pose significant challenges in treatment due to their multidrug-resistant nature. Ceftolozane/tazobactam (C/T) is a novel antimicrobial agent that has shown promising activity against these pathogens. However, there is limited data

available on its susceptibility against these resistant isolates in Korea. The aim of this study is to determine the in vitro susceptibility of C/T against CRE and CRPA isolates in a tertiary hospital in Korea.

**Methods:**

Blood isolates of CRE and CRPA were collected from a tertiary hospital between September 2017 and September 2022. A carbapenemase test was performed using NG test CARBA-5. For non-carbapenemase-producing CRE and CRPA, the minimum inhibitory concentration of C/T was determined using the Etest and interpreted according to the CLSI breakpoints.

**Results:**

A total of 120 blood isolates, including 55 CRE and 65 CRPA, were analyzed. Carbapenemase-producing isolates were found in 60.0% of CRE and 27.7% of CRPA samples. Among the CRE isolates, KPC (50.9%), NDM (7.3%), and OXA-48-like (1.8%) carbapenemases were identified, while NDM (21.5%), IMP (3.1%), and VIM (3.1%) were found in CRPA isolates. For the non-carbapenemase-producing isolates, the susceptible, intermediate, and resistant rates to C/T were 9.1%, 7.3%, and 23.6%, respectively, for CRE, and 67.7%, 3.1%, and 1.5%, respectively, for CRPA.

**Conclusion:**

This study provides important information on the in vitro susceptibility of C/T against CRE and CRPA isolates in Korea. The findings will help guide empirical antibiotic therapy and improve patient outcomes. Further studies are needed to evaluate the clinical efficacy of C/T against these resistant pathogens in Korea.

**Disclosures:**

**All Authors**: No reported disclosures

